# Electrosynthesis-Induced Pt Skin Effect in Mesoporous Ni-Rich Ni–Pt Thin Films for Hydrogen Evolution Reaction

**DOI:** 10.1021/acsami.4c09288

**Published:** 2024-08-02

**Authors:** Konrad Eiler, Salvador Pané, Max Döbeli, Arnold Müller, Christof Vockenhuber, Jordi Sort, Eva Pellicer

**Affiliations:** † Departament de Física, Universitat Autònoma de Barcelona, 08193 Bellaterra, Spain; ‡ Institute of Robotics and Intelligent Systems (IRIS), ETH Zurich, CH-8092 Zurich, Switzerland; § Laboratory of Ion Beam Physics, ETH Zurich, Otto-Stern-Weg 5, CH-8093 Zurich, Switzerland; ∥ Institució Catalana de Recerca i Estudis Avançats (ICREA), Pg. Lluís Companys 23, 08010 Barcelona, Spain

**Keywords:** hydrogen, electrodeposition, thin films, mesoporous materials, water splitting, Pt skin effect, ion beam analysis

## Abstract

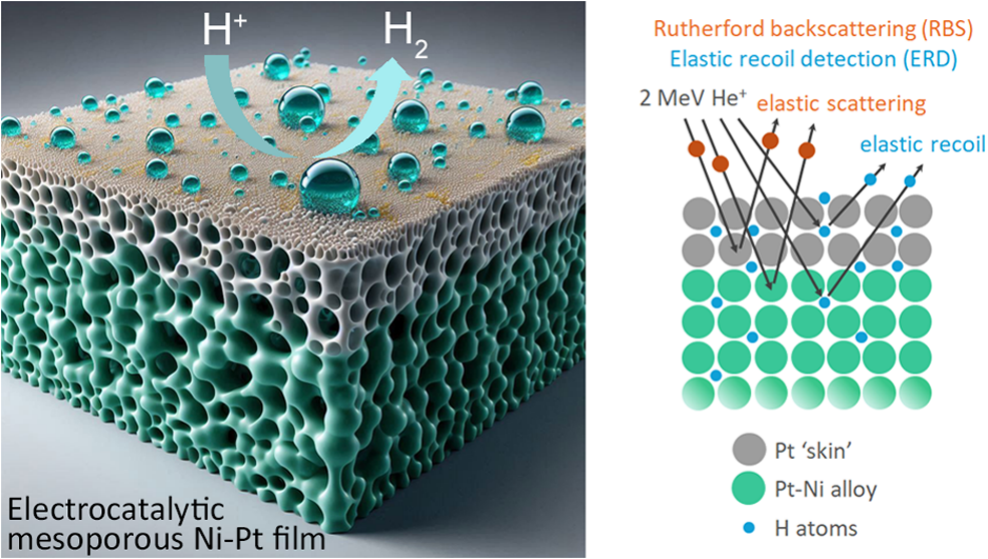

A Pt skin effect, i.e., an enrichment of Pt within the first 1–2 nm from the surface, is observed in as-prepared electrodeposited Ni-rich Ni–Pt thin films. This effect, revealed by Rutherford backscattering (RBS), is present for both dense thin films and mesoporous thin films synthesized by micelle-assisted electrodeposition from a chloride-based electrolyte. Due to the Pt skin effect, the Ni-rich thin films show excellent stability at the hydrogen evolution reaction (HER) in acidic media, during which a gradient in the Pt/Ni ratio is established along the thickness of the thin films, while the activity at the HER remains unaffected by this structural change. Further characterization by elastic recoil detection with He ions analysis shows that hydrogen profiles are similar to those of Pt: a surface hydrogen peak coincides with the Pt skin, and a gradient in hydrogen concentration is established during HER in acidic media, together with a considerable uptake in hydrogen. A comparative study shows that in alkaline media, hydrogen evolution has little to no effect on the structural properties of the thin films, even for much longer times of exposure. The mesoporous thin films, in addition to their higher efficiency at HER compared to dense thin films, also show lower internal stress, as determined by Rietveld refinement of grazing incidence X-ray diffraction patterns. The latter also reveal a fully single-phase and nanocrystalline structure for all thin films with varying Ni contents.

## 1 Introduction

With the global efforts for fully renewable energies and responsible use of resources, electrocatalysts for water splitting are in focus with the need to minimize the use of platinum group metals (PGM). Nickel, as an abundant resource, is predestined as an electrocatalyst for use in hydrogen production due to its generally high catalytic activity, but suffers from compromised stability, especially in acidic media.^1^

Mesoporous Ni-rich Ni–Pt thin films, synthesized by micelle-assisted electrodeposition, have been previously reported to show excellent performance at hydrogen evolution reaction (HER) in acidic media, including sufficient long-term stability.^2,3^ Due to their high Ni content, both performance and stability in acidic media are at least surprising, and this is the motivation for a more in-depth structural analysis of the thin films. A surface enrichment in Pt, known as the Pt skin effect, is a known phenomenon that has been reported for various systems after subjection to electrocatalytic reactions, especially for bimetallic Pt–M (M being Ni, Co, Fe, Ti or V) alloys.^4^ Naturally, such Pt skin is electrochemically more stable in acidic media than the underlying, less noble Pt–M alloy and thus able to protect it from degradation or preferential leaching. Greeley and Nørskov estimated the dissolution potential of Pt on the Pt skin surfaces of Pt–M alloys to be significantly higher than on pure Pt.^5^

For the oxygen reduction reaction (ORR), Pt–M catalysts with Pt skin layer show reportedly higher electrocatalytic activity compared to pure Pt.^6^ In most cases, the Pt skin either establishes electrochemically during cycling or ORR^7^ or is formed by thermal treatment as part of the synthesis.^8,9^ Fortunelli et al. investigated the case of Ni_7_Pt_3_ in a computational study, suggesting that the enhanced activity at the ORR is a result of surface dealloying, resulting in a rather defective surface structure, which is more favorable toward the ORR compared to a regular, pure Pt surface.^10^ Alternatively, an enhanced electrocatalytic activity is being related to changes in the electronic structure of Pt and weaker oxygen binding energies due to the underlying transition-metal species.^11,12^

Effects of long-term hydrogen evolution are mostly related to the media in which electrocatalysis takes place. The role of hydrogen itself, although it is the main element in focus for water splitting, is mostly neglected, probably because the most common surface analysis (XPS, EDX) and chemical analysis techniques (ICP) are not sensitive to hydrogen. In consequence, little is known about hydrogen contents in common electrocatalysts. In the case of Ni-based catalysts, having an idea on their hydrogen content is especially valuable since hydrogen can easily dissolve interstitially in the face-centered cubic (fcc) lattice and may lead to hydrogen embrittlement;^13^ however, the absorption of hydrogen is reversible.^14^ In fact, strong hydrogen evolution at Ni electrodes in alkaline media has been used to study the incorporation of interstitial hydrogen and the formation of Ni hydrides by X-ray diffraction (XRD).^15^ Interestingly, Faid et al. showed that while hydrogen is incorporated during HER, surface oxides and hydroxides of Ni are eliminated at large cathodic current densities.^16^

In this work, an in-depth structural characterization is performed for Ni-rich Ni–Pt thin films with different compositions and in both mesoporous and dense configurations. The occurrence of a Pt skin layer and its behavior during HER in both acidic and alkaline media are investigated, discussing the effects of composition, porosity, and structural parameters. In addition, the role of elementary hydrogen in the thin films and the effects of the HER with respect to hydrogen content are investigated.

## 2 Experimental Section

The micelle-assisted electrodeposition for the synthesis of mesoporous Ni-rich Ni–Pt thin films as well as the deposition of the continuous Ni–Pt thin films is extensively reported elsewhere.^2^ Briefly, the deposition was carried out potentiostatically in a three-electrode cell from an electrolyte of 100 mL in volume containing 200 mM NiCl_2_, 3 mM Na_2_PtCl_6_, 200 mM H_3_BO_3_, 25 mM NH_4_Cl, and 10 g/L Pluronic P-123 at pH 2.7. A range of thin films with different Ni–Pt ratios are synthesized by depositing at different potentials vs Ag|AgCl (3 M KCl), using Pt wire as a counter electrode, onto Si/Ti/Cu substrates with a working area of 1.2 cm^2^. Deposition times were 45 s at −1.2 V, 90 s at −0.9 V, and 180 s at −0.7 V for both mesoporous and dense thin films, while the electrolyte was agitated using a magnetic stir bar of 30 mm length at 100 rpm. The continuous (dense) thin films are obtained when the micelle-forming surfactant Pluronic P-123 is omitted from the electrolyte. The films were then rinsed with acetone, ethanol, and water. For the mesoporous films, the surfactant occupying the pores was dissolved by immersion in ethanol for 10 min.

The composition of the thin films in terms of Ni/Pt ratio was determined by inductively coupled plasma-optical emission spectroscopy (ICP-OES) on a PerkinElmer Optima 4300DV, following their dissolution in aqua regia.

Scanning electron microscopy (SEM) was performed on a Zeiss Merlin electron microscope using an InLens detector with an acceleration voltage of 1 kV.

Grazing incidence X-ray diffraction (GIXRD) was performed using a Malvern-PANalytical X’pert Pro MRD diffractometer in a 2θ range of 39–102° for the first experiment. Since no signals were either obtained or expected in the range of 54–68°, this region was omitted for subsequent measurements. For the determination of lattice parameter, crystallite size, and microstrain, Rietveld refinement was conducted using software MAUD.^17^

Electrocatalytic activity at HER was performed in a three-electrode cell using an Ag|AgCl (3 M KCl) reference electrode and a carbon rod (in acidic media) or Pt wire (in alkaline media) as the counter electrode, connected to an Autolab 302N potentiostat. Dense and mesoporous thin films were polarized at −0.3 V vs Ag|AgCl for 30 min in 0.5 M H_2_SO_4_ for subsequent chemical characterization by Rutherford backscattering (RBS) and elastic recoil detection (ERD). For comparison, the films were also subjected to HER in alkaline 1 M KOH. Since HER in alkaline media was expected to yield little effect, it was carried out for a prolonged time of 24 h and at a constant geometric current density of −10 mA/cm^2^, which is higher than the current density obtained at HER in the acidic media. All measured potentials were converted to a reversible hydrogen electrode (RHE).

RBS and He-ERD were performed using a 2 MeV ^4^He^+^ ion beam with a backscattering angle of 167.5° (RBS) and a symmetric forward scattering angle of 15° relative to the sample surface (He-ERD), respectively. The simulations of the RBS and He-ERD spectra were performed using RUMP and SIMNRA code.^18,19^ For the hydrogen depth profiles obtained by He-ERD, a mica standard with 9.5 atom % H was used for normalization.

## 3 Results and Discussion

**Figure 1:**
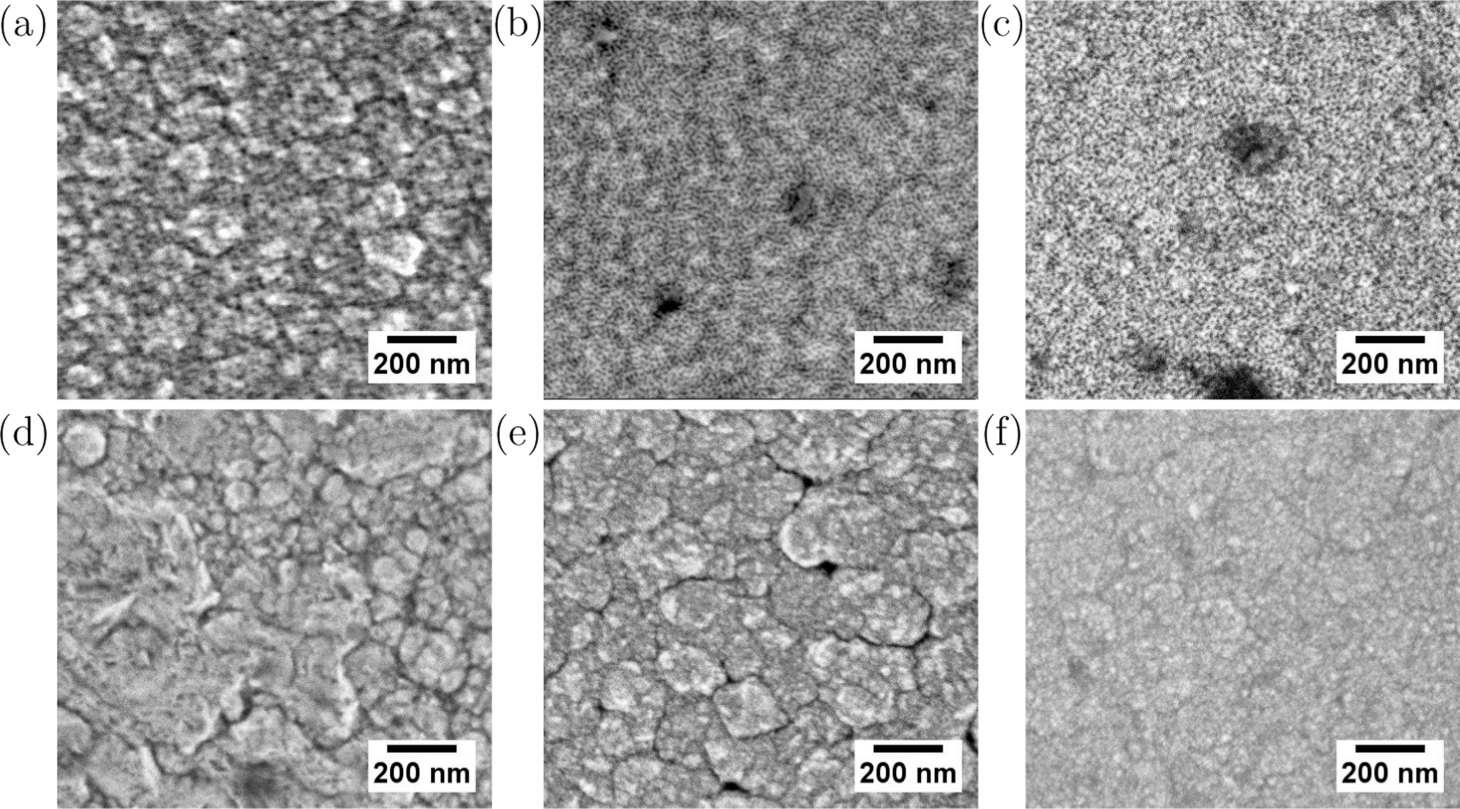
SEM micrographs of mesoporous Ni_98_Pt_2_ (a), Ni_92_Pt_8_ (b), Ni_76_Pt_26_ (c), and dense Ni_98_Pt_2_ (d), Ni_91_Pt_9_ (e), and Ni_79_Pt_21_ (f) thin films.

From the electrodeposition at three different reduction potentials of mesoporous and dense Ni–Pt thin films, three porous/dense pairs with comparable composition are obtained, allowing investigation of both the effects of composition as well as those of mesoporosity. As determined by ICP-OES, the compositions of the mesoporous thin films are Ni_98_Pt_2_, Ni_92_Pt_8_, and Ni_76_Pt_24_, their dense counterparts being Ni_98_Pt_2_, Ni_91_Pt_9_, and Ni_79_Pt_21_, respectively. The porous films show a homogeneously mesoporous structure with a higher roughness related to grain growth for Ni_98_Pt_2_ (Figure 1). With respect to our earlier work, the mesoporous morphology is successfully reproduced.^2^ Ni_92_Pt_8_ and Ni_76_Pt_24_ films show equally smooth surfaces, while for Ni_98_Pt_2_, the previously observed grain structure is less pronounced. The dense films show a notably higher grain size, with occasional voids between grains, and with the tendency to have a smoother appearance at higher Pt contents. Dense Ni_98_Pt_2_ shows highly irregular grain growth in terms of size and morphology.

**Figure 2:**
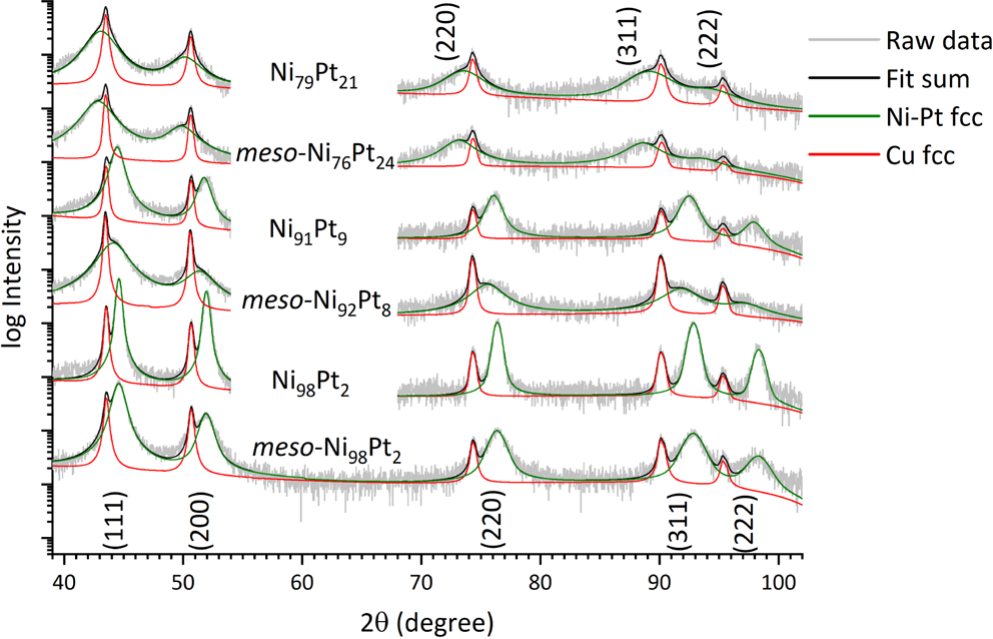
XRD patterns of mesoporous and dense Ni–Pt films as deposited, including the fitted Ni–Pt and Cu fcc phases.

All Ni–Pt thin films, regardless of their porous or continuous morphology, show a fully single-phase character with an fcc structure. All expected diffraction peaks for fcc in the measurement range are present, namely, (111), (200), (220), (311), and (222), for both the Ni–Pt thin films as well as the Cu seed layer, which has a comparable cell parameter *a* (Figure 2). There is no apparent texture observed in the thin films. The peak positions of the Ni–Pt phase change with composition due to the change in cell parameters according to Vegard’s law.^20^

Comparing the structural parameters obtained by Rietveld refinement, the crystallite size *d* is lower for mesoporous thin films compared to their dense counterparts (Table 1). This observation suggests that the mesopores limit crystal growth during the synthesis. In addition, for both mesoporous and dense films, the crystal size is refined when the Pt content is higher, which is in accordance with the observation from SEM, which reveals the presence of finer grains at higher Pt contents. Assuming a fully single-phase character, seconded by the fact that the Ni–Pt system has a negative enthalpy of mixing and therefore no segregation is expected, the obtained cell parameters correspond well with our earlier work and data from the literature.^21−23^ Regarding the microstrain values determined for each configuration, these are generally lower in the mesoporous films, suggesting that the porous structure allows the metal lattice to relax and mitigates the buildup of stress throughout the thickness. In this case, the Ni–Pt films with porous structure are protected against strain-induced cracking, thus revealing a second advantage, in addition to an increased surface area. An exception is observed for mesoporous Ni_98_Pt_2_, where the higher microstrain ϵ may be caused by the predominant grain structure and growth, which counteracts the stress relief related to the mesoporosity (cf. Figure 1a).

**Table 1:** Summarized Results for the Electrodeposited Ni–Pt Thin Films

	*E*_dep_	*t*_dep_	*a*	*d*	ϵ
sample	[V vs Ag|AgCl]	[s]	[Å]	[nm]	[%]
*meso*-Ni_98_Pt_2_	–1.2	45	3.53	12.4	0.37
Ni_98_Pt_2_	–1.2	45	3.53	38.4	0.22
*meso*-Ni_92_Pt_8_	–0.9	90	3.56	4.4	0.01
Ni_91_Pt_9_	–0.9	90	3.54	11.6	0.23
*meso*-Ni_76_Pt_24_	–0.7	180	3.66	4.1	0.03
Ni_79_Pt_21_	–0.7	180	3.64	4.8	0.75

**Figure 3:**
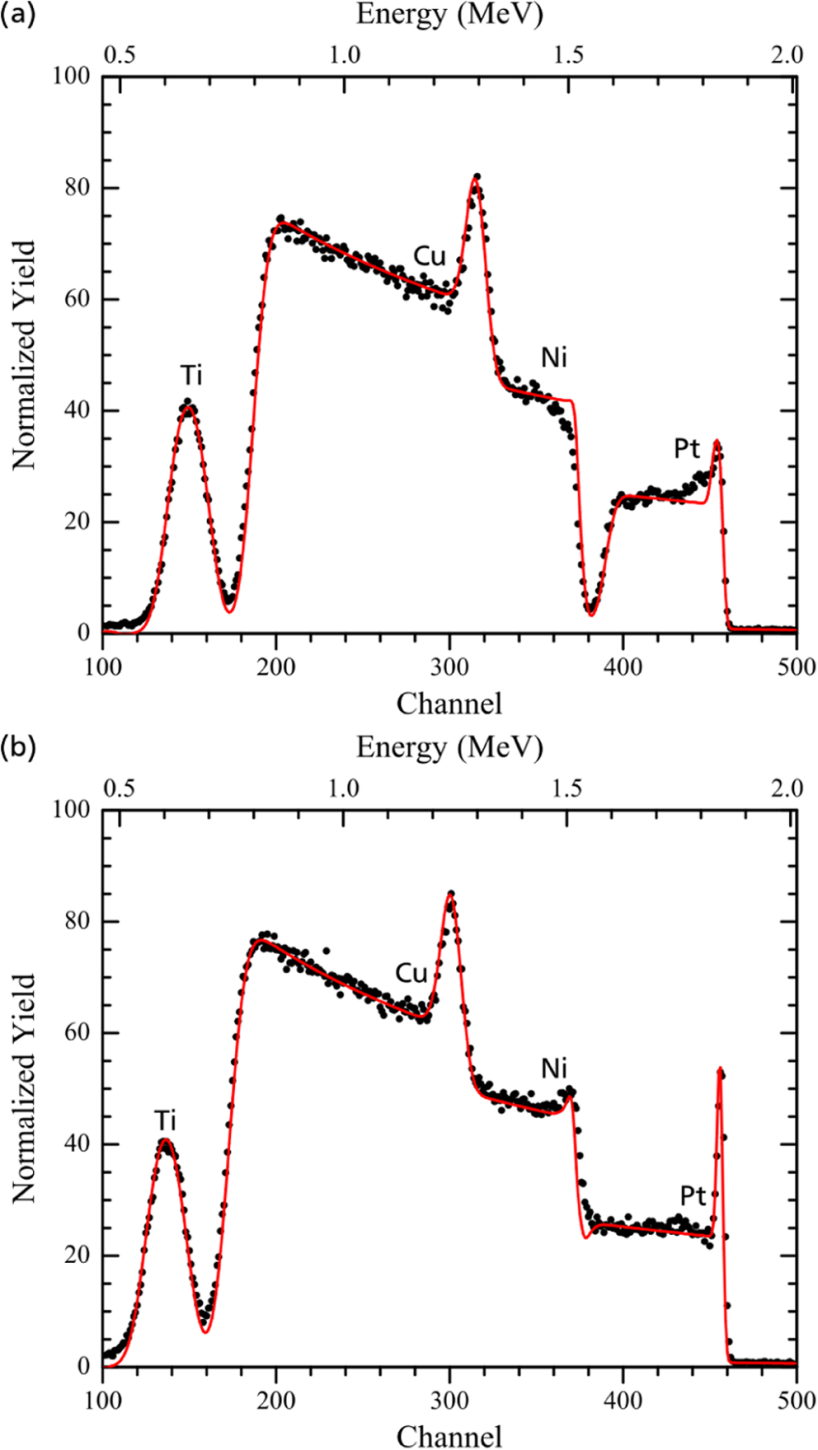
2 MeV He^+^ RBS spectrum of as-deposited mesoporous Ni_92_Pt_8_ (a) and dense Ni_91_Pt_9_ (b) thin films. Markers indicate the measured yields, while the continuous line shows the simulation. In addition to Ni and Pt of the electrodeposited film, Cu and Ti from the underlying substrate layers are present in the spectra.

The presence of a Pt-rich surface layer is evident for as-deposited dense and mesoporous films in the corresponding RBS spectra (Figure 3). The layer is evidenced by the higher relative intensity in the Pt signal at the Pt edge and confirmed by the simulation to correspond to 2.2 nm of pure Pt in the case of dense Ni_91_Pt_9_. For the mesoporous thin film, this signal at the Pt edge is less sharp, most probably due to the porosity on the surface, which leads to a larger spread of the signal (Figure 3a). The formation of the Pt skin during the synthesis is most likely due to spontaneous redox replacement of Ni by Pt once the electrodeposition process is finished and the potential returns to open circuit potential (OCP),^24,25^ even though the samples remain in this condition for a very short time of only 2–3 s since they are immediately removed from the electrolyte and rinsed in water. In aggressive media, such a short time scale is sufficient to cause dealloying of the less noble metal and the appearance of a significant surface peak of the noble metal.^26,27^ Another possible reason for the formation of the Pt skin at the end of the electrodeposition process is the leaching of Ni back into the electrolyte due to its acidic pH of 2.7. Additionally, electroless reduction of Pt may take place on the surface as soon as the electrode returns to OCP. Since electroless deposition can often be triggered by displacement, the agitation of the electrolyte alone may be able to produce a very thin Pt surface layer in a very short time, even in the absence of a reducing agent.^28,29^ It is likely that the Pt skin forms by following a combination of the mentioned mechanisms.

**Figure 4:**
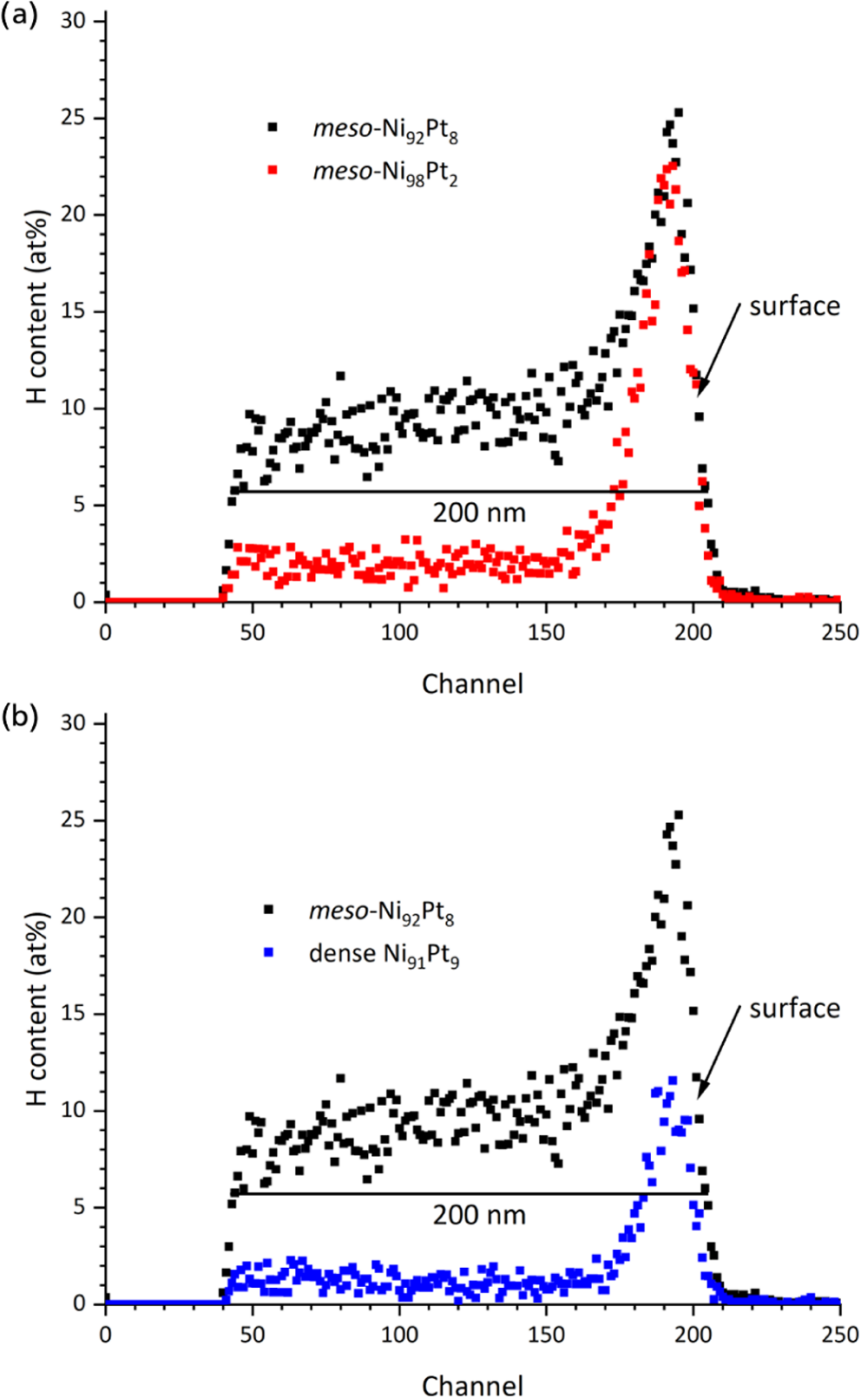
2 MeV He^+^ ERD spectra for hydrogen, comparing as-deposited mesoporous thin films Ni_92_Pt_8_ and Ni_98_Pt_2_ (a), and comparing as-deposited mesoporous Ni_92_Pt_8_ with dense Ni_91_Pt_9_ (b). Note that the information depth is 200 nm, which does not represent the film thickness.

Concerning the hydrogen distribution, different observations are made regarding the as-deposited thin films. First of all, all thin films exhibit hydrogen enrichment at the surface. Second, the subsurface hydrogen content is lower when the Pt content is lower (Figure 4a). This may be related to the previously observed lower deposition rates for thin films with higher Pt content, leading to a higher probability for the incorporation of hydrogen through the hydrogen evolution side reaction.^21^ At the same time, higher Pt contents may enhance the hydrogen evolution side reaction during the deposition process, even though the deposition potentials are less negative. Note that thin films with higher Pt contents also show higher concentrations of oxygen.^2^ Finally, the as-prepared dense Ni–Pt films show lower hydrogen contents than their mesoporous counterparts; however, the hydrogen content in the dense thin films remains considerable (approximately 2 atom %, Figure 4b). In view of the non-negligible hydrogen contents, these may have an effect on the X-ray diffractograms, leading to a widening of the peaks^15^ and an underestimation of the mean crystallite size (cf. Figure 2).

**Figure 5:**
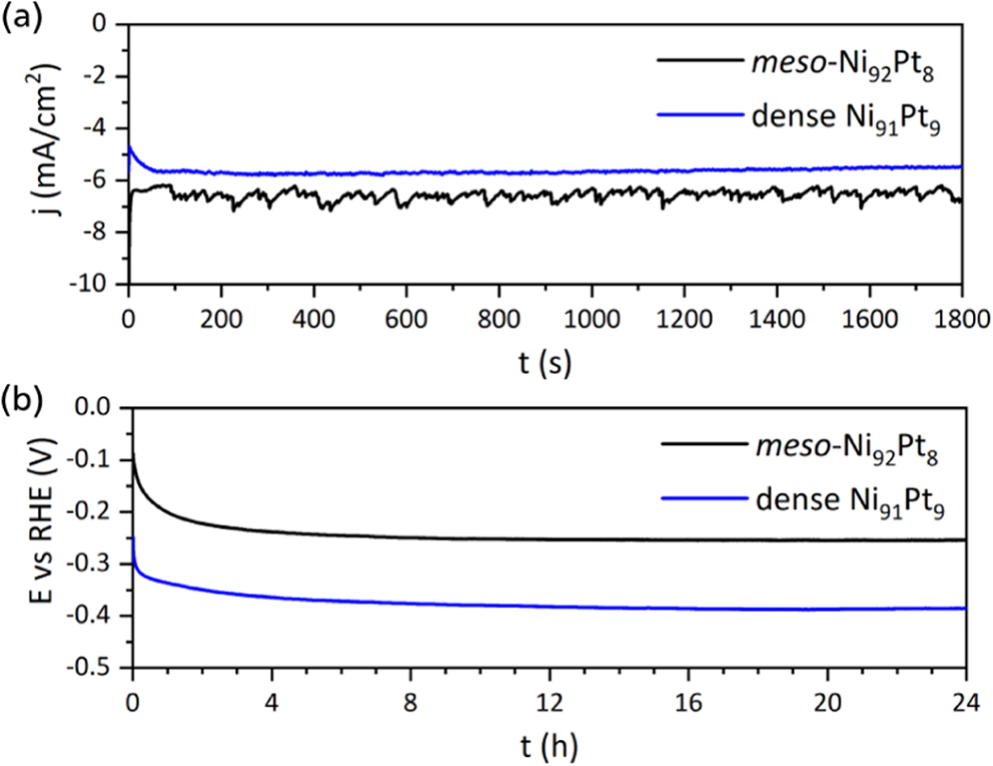
Hydrogen evolution for 30 min at −90 mV vs RHE in 0.5 M H_2_SO_4_ (a) and for 24 h at −10 mA/cm^2^ in 1.0 M KOH (b), for mesoporous Ni_92_Pt_8_ and dense Ni_91_Pt_9_ thin films.

Both in acidic and alkaline media, the behavior of the Ni–Pt films at constant hydrogen evolution is rather similar. In acidic media, at a constant overpotential of −90 mV vs RHE, the current density equilibrates for about 1 min, after which it stays constant. As expected, the mesoporous Ni_92_Pt_8_ thin film is more active at HER than dense Ni_91_Pt_9_, achieving a higher geometric current density. Due to the porous structure and possible temporary trapping of hydrogen bubbles on the surface leading to temporary changes in the surface area, the measured current density fluctuates (Figure 5a). In alkaline media, at a constant current density of −10 mA/cm^2^, the potential reaches equilibrium after a few hours, while the largest part of the equilibration takes place during the first hour. Again, the mesoporous thin film shows superior performance with a lower overpotential for HER (Figure 5b).

**Figure 6:**
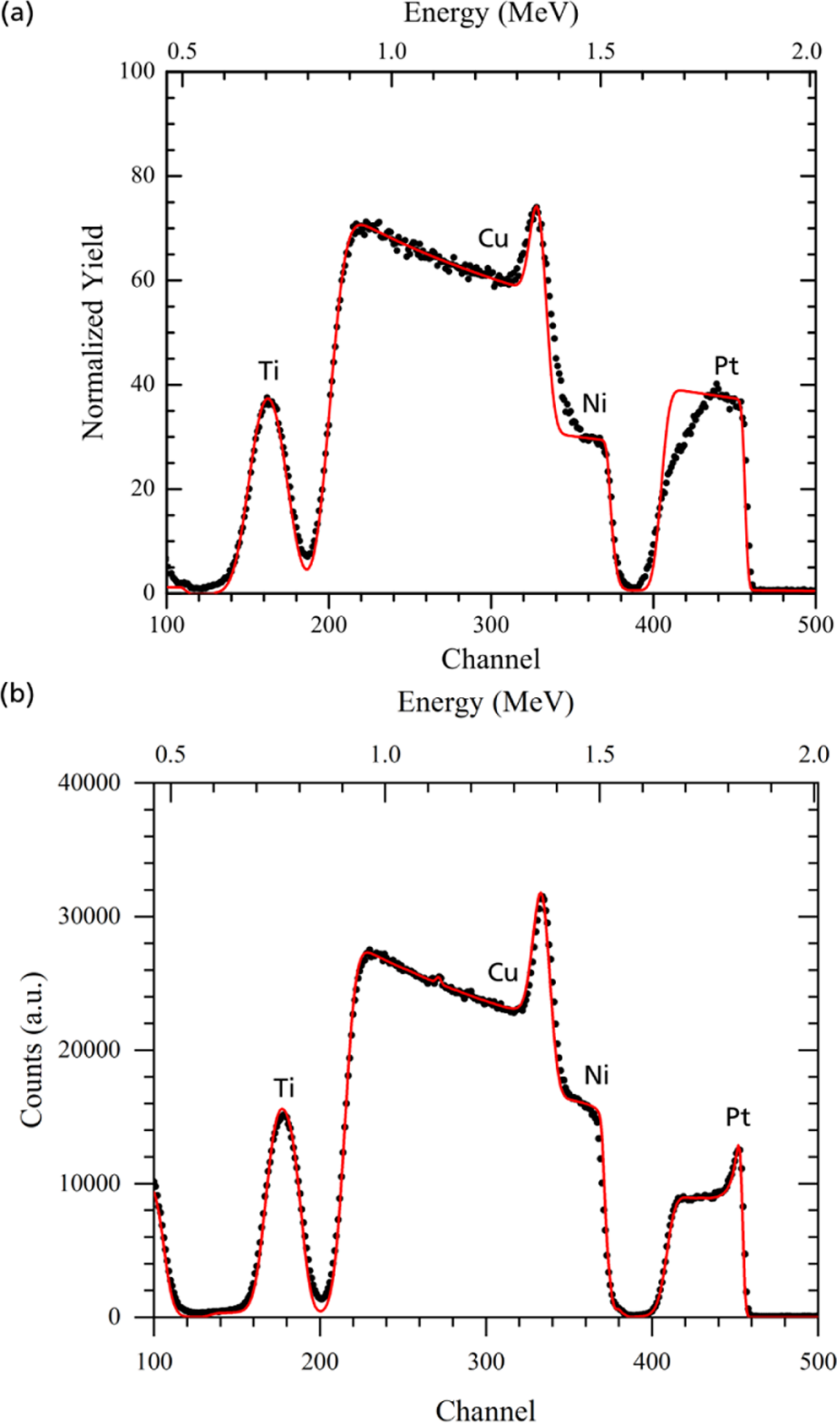
2 MeV He^+^ RBS spectrum of a mesoporous Ni_92_Pt_8_ thin film after 30 min at a potential of −90 mV vs RHE in 0.5 M H_2_SO_4_ (a) and after 24 h at −10 mA/cm^2^ in 1 M KOH (b). Markers indicate the measured yields, while the continuous line shows the simulation. Note that the simulation in panel (a) considers a Ni–Pt film with constant composition, while the measurement indicates a decreasing Pt/Ni ratio with depth.

**Figure 7:**
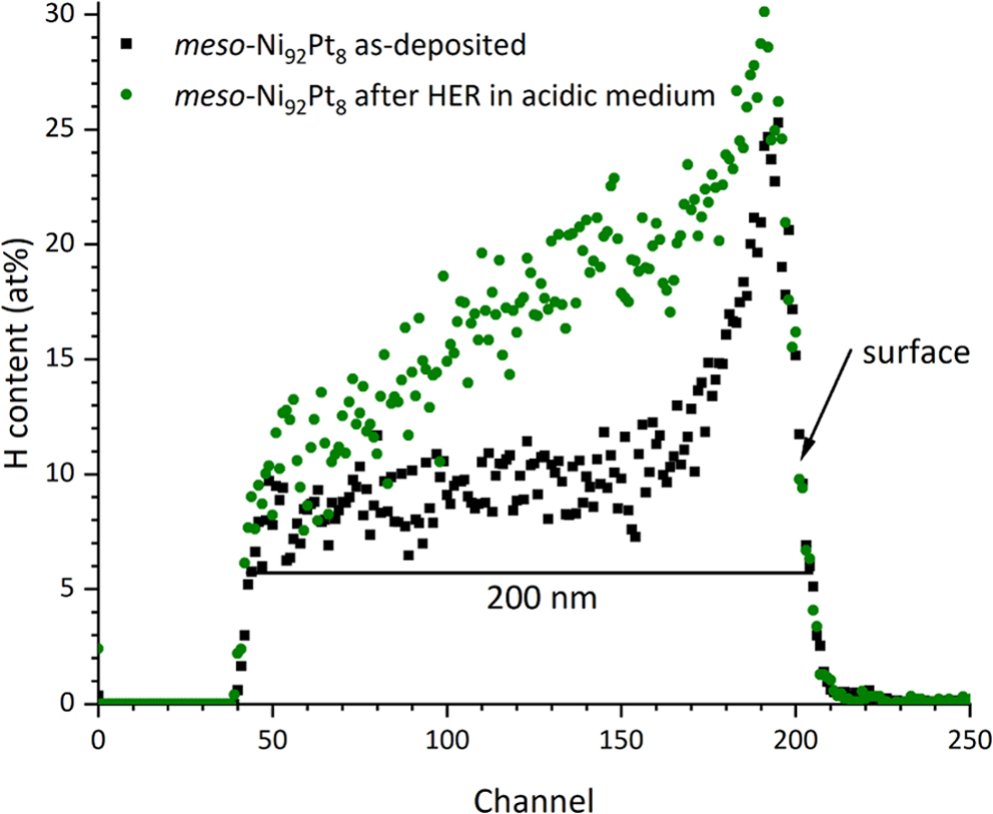
2 MeV He^+^ ERD spectra for hydrogen, comparing mesoporous thin films Ni_92_Pt_8_ before and after HER for 30 min at −90 mV vs RHE in 0.5 M H_2_SO_4_.

After 30 min of constant hydrogen evolution in acidic media, a compositional gradient is established throughout the Ni–Pt films, where the Pt content continually increases toward the surface and is constant in the near-surface region (Figure 6a). In this way, the clear distinction between the previously observed Pt skin and the underlying Ni–Pt layer has disappeared. The hydrogen distribution after HER shows a considerable hydrogen uptake, taking a similar shape as the Pt distribution and showing increased H concentration throughout the entire information depth of 200 nm, which is close to the full film thickness of 215 nm, as determined by RBS (Figure 7). For the dense configuration after HER in acidic media, the observations are similar to those of the mesoporous films, with a compositional gradient of Ni and Pt throughout the thin films and a considerable hydrogen uptake (not shown).

After HER in alkaline media, even after 24 h, the RBS profile of mesosporous Ni_92_Pt_8_ remains nearly identical to the one obtained on the as-deposited film (Figure 6b). Likewise, a significant uptake in hydrogen was not observed by ERD. The observations made in acidic media might suggest that the restructuring of the surface takes place by Pt surface diffusion via hydrogen adatoms, which may progress throughout the thickness over the surface of the mesopores.^30^ Considering that these effects are not observed after HER in alkaline media, surface diffusion can be excluded as the main driving force; in addition, this surface diffusion is not expected to be directional during the HER. Instead, the observations must be related to the acidic media itself and suggest that both the hydrogen uptake as well as the compositional gradient throughout the entire film thickness are a result of the penetration of the electrolyte through the porous structure rather than a subsurface diffusion process that would not require contact with the electrolyte.

In fact, the predominant processes responsible for establishing the compositional gradient of Ni and Pt should be similar to the ones discussed as being responsible for the formation of the Pt skin during the synthesis. A partial dissolution of metallic species may occur, even during cathodic polarization, and preferentially of the less noble Ni species. In our previous work, both Ni and Pt were detected in acidic media after HER.^2^ In contrast to dealloyed films, which display similar RBS profiles of noble metals after chemical or electrochemical attack,^26,27^ a significant decrease in film thickness is not appreciated due to the redeposition of dissolved species at cathodic potentials. Close to the surface, dissolved Ni species can escape more easily, whereas below the surface, Ni ions are more easily trapped in the pores, and the probability for redeposition is higher. Pt, if dissolved, should redeposit faster since its deposition potential is less negative;^2^ therefore, its diffusion into the electrolyte is hindered. In addition, in the presence of hydrogen gas and, more importantly, more reactive hydrogen adatoms as an intermediate species of the HER,^31^ Ni may redeposit in the form of hydrides or hydroxides. The latter, together with the direct formation of hydrides due to HER,^32^ account for the considerable hydrogen uptake. A more defective nanostructure caused by partial dissolution may also facilitate the incorporation of atomic hydrogen and can favor the above-mentioned hydrogen-enabled surface diffusion, which should increase surface mobility and can thus facilitate the restructuring through dissolution and redeposition.

Interestingly, the changes observed by both RBS and ERD do not reflect at all in the chronoamperometric measurements (cf. Figure 5a). This may be because the observed changes mostly affect subsurface regions of the thin films without a major change in surface composition. In our earlier work, a slight activation of the Ni–Pt surfaces was observed by X-ray photoelectron spectroscopy (XPS) after 200 linear sweep voltammetries in 0.5 M H_2_SO_4_, where the surfaces showed higher relative amounts of metallic Pt and metallic Ni after HER.^2^

## 4 Conclusions

The electrodeposition of dense and mesoporous Ni–Pt thin films leads to a self-induced Pt skin effect, as evidenced by RBS. The Pt skins are found to be nearly pure Pt, of 1–2 nm in thickness, after which the Pt/Ni ratio of the films is constant. The formation of the Pt skin may be triggered due to galvanic replacement of Ni by Pt, electroless deposition of Pt by displacement, or the dissolution (preferential leaching) of Ni back into the acidic electrolyte, as the electrodeposition process is terminated. Interestingly, a strong hydrogen surface peak coincides with the Pt skin, as observed by ERD.

During HER in acidic media, a gradient in the Pt/Ni ratio is established throughout the entire thickness of the films, in a way that the Pt content increases toward the surface. This observation is rather surprising, since the electrocatalytic behavior observed by chronoamperometry does not show any changes apart from an initial equilibration. In analogy to Pt, a significant amount of hydrogen is incorporated in the films, establishing a gradient that roughly follows the profile of Pt. The structural changes seem to stabilize the thin films in acidic media, the enrichment of Pt toward the surface guaranteeing both chemical stability and electrocatalytic activity. In contrast, HER in alkaline media, even for a much longer duration, does not lead to any structural subsurface changes in the thin films, and the Pt skin remains equal to the as-prepared state. Thus, the compositional restructuring and hydrogen uptake in acidic media are driven by the dissolution and redeposition of the metallic species rather than by surface or bulk diffusion processes. Both in acidic and alkaline media, the mesoporous thin films show superior performance over their dense counterparts due to their higher specific surface area.

All thin films show a single-phase, nanocrystalline character by GIXRD. The mesoporous films, obtained by micelle-assisted electrodeposition, show lower crystallite sizes than their dense counterparts, in addition to generally lower microstrain (cf. Table 1), indicating better stability and lower susceptibility to cracking. The mesoporosity is homogeneously distributed with pore sizes on the order of 10 nm.
